# The Compliance of the Upper Critical Field in Magic-Angle Multilayer Graphene with the Pauli Limit

**DOI:** 10.3390/ma16010256

**Published:** 2022-12-27

**Authors:** Evgueni F. Talantsev

**Affiliations:** M. N. Miheev Institute of Metal Physics, Ural Branch, Russian Academy of Sciences, 18, S. Kovalevskoy St., 620108 Ekaterinburg, Russia; evgeny.talantsev@imp.uran.ru or evgeny.f.talantsev@gmail.com; Tel.: +7-912-676-0374

**Keywords:** Pauli limiting field, superconducting energy gap, upper critical field

## Abstract

The Pauli limiting field represents a fundamental magnetic field at which the superconducting state collapses due to the spin-paramagnetic Cooper pair-breaking effect. Cao et al. (*Nature* **2021**, *595*, 526) reported that the magic-angle twisted trilayer graphene (MATNG, *N* = 3) exhibits the upper critical field which exceeds the Pauli limiting field by two to three times. This observation was interpreted as a violation of the Pauli-limiting field in MAT3G. Similar conclusions were recently reported by the same research group in MATNG (*N* = 4, 5) superlattices (Park, J.M. et al. *Nat. Mater.* **2022**, *21*, 877). Here, we point out that Cao et al. (*Nature* **2021**, *595*, 526) calculated the Pauli limiting field by the use of reduced form (to the weak-coupling limit) of full equation of the theory of the electron–phonon-mediated superconductivity. Considering that in the same paper, Cao et al. (*Nature* **2021**, *595*, 526) reported that MATNGs are strong coupled superconductors, we calculate the Pauli limiting field for a strong coupled case and show that the observed upper critical fields in MATNGs comply with the Pauli limit. This implies that there is no violation of the Pauli limiting field in the Moiré multilayer graphene superlattices.

## 1. Introduction

In order to define the fundamental upper limit for the magnetic field at which the superconducting state collapses, Clogston [1] and Chandrasekhar [2] introduced the so-called Pauli limiting field, $B_{p}$, i.e., the field at which the Cooper pairs break due to the spin-paramagnetic effect. In the theory of electron–phonon-mediated superconductivity, the ground state Pauli limiting field, $B_{p}\left(0\right)$, is given by the equation [3,4]:

(1) $$B_{P}\left(0\right)=\frac{\Delta \left(0\right)}{\sqrt{g}\times \mu_{B}}\times \left(1+\lambda_{e-ph}\right)$$

where $\Delta \left(0\right)$ is the ground state amplitude of the superconducting energy gap, $\mu_{B}$ is the Bohr magneton, g is the Lande factor (if spin-orbit scattering is negligible, then g=2), and $\lambda_{e-ph}$ is the electron–phonon coupling constant. By its definition, the experimentally observed upper critical field, $B_{c2}\left(T\right)$ (i.e., the field at which the superconducting state collapses in experiment), should not be higher than $B_{p}\left(T\right)$:

(2) $$B_{c2}\left(T\right)\leq B_{P}\left(0\right)$$

However, recently Cao et al. [5] reported a different result for magic-angle twisted trilayer graphene (MATTG) [5]. Cao et al. [5] measured the parallel upper critical field, $B_{c2,||}\left(T\right)$ (i.e., the upper critical field when the external magnetic field is applied in parallel direction to the film surface), in magic-angle twisted trilayer graphene (MATTG). Cao et al. [5] utilized the simplest equation [6,7] of the Ginzburg–Landau (GL) theory to deduce the ground-state parallel upper critical field $B_{c2,||}\left(0\right)$ from $B_{c2,||}\left(T\right)$ datasets in MATTG:

(3) $$B_{c2,||}\left(0\right)=\frac{B_{c2,||}\left(T\right)}{\sqrt{1-\frac{T}{T_{c}}}}$$

Based on deduced $B_{c2,||}\left(0\right)$ values, Cao et al. [5] reported that MAT*N*G (*N* = 3) exhibits $B_{c2,||}\left(0\right)$ which is 2–3 times larger than the Pauli limiting field, $B_{P}\left(0\right)$:

(4) $$2\times B_{P}\left(0\right)≲B_{c2,||}\left(T\to 0\;K\right)≲3\times B_{P}\left(0\right)$$

This finding is in a good accord with the majority of recent papers on atomically thin superconductors (published by many research groups [8,9,10,11]) where similar observations to Equation (4) were also reported.

Here, we stress that the Pauli limiting field, $B_{P}\left(0\right)$, in references [5,8,9,10,11] was calculated by the use of reduced (to *s*-wave weak-coupling limit of the Bardeen–Cooper–Schrieffer theory of superconductivity [12]) form of Equation (1), which is:

(5) $$B_{P,BCS}\left(0\right)=1.86\times T_{c}.$$

Equation (5) can be obtained from Equation (1) if the BCS weak-coupled limiting values [12]: $\lambda_{e-ph}=0$, g=2, and $\frac{2\Delta \left(0\right)}{k_{B}T_{c}}=3.53$ (where $k_{B}$ is the Boltzmann constant, $T_{c}$ is the superconducting transition temperature) will substitute in Equation (1):

(6) $$B_{P,BCS}\left(0\right)=\frac{\Delta \left(0\right)}{\sqrt{g}\times \mu_{B}}\times \left(1+\lambda_{e-ph}\right)=\frac{1}{\sqrt{8}}\times \frac{k_{B}}{\mu_{B}}\times \left[\frac{2\Delta \left(0\right)}{k_{B}T_{c}}\right]\times T_{c}=1.86\times T_{c}$$

Based on this, what was actually observed in Refs. [5,8,9,10,11] is:

(7) $$3.7\times T_{c}\leq B_{c2,||}\left(T\to 0\;K\right)\leq 5.6\times T_{c}$$

Cao et al. [5,8] introduced the so-called the Pauli violation ratio (PVR), defined as:

(8) $$PVR=\frac{B_{c2,||}\left(T\to 0\;K\right)}{B_{P,BCS}\left(0\right)}=\frac{B_{c2,||}\left(T\to 0\;K\right)}{1.86\times T_{c}}$$

However, the introduction of this parameter [5,8] is not necessary, because theoretical concepts describing the superconducting state [3] are based on well-established universal characteristic values (for instance, on the constant of the interaction strength, $\lambda_{e-ph}$), which have universal meaning across superconducting and normal properties of the material. As we show below, the amplitude of the upper critical field, $B_{c2,||}\left(T\to 0\;K\right)$, can be calculated based on the $\lambda_{e-ph}$ and there are no violations in calculated $B_{c2,||}\left(T\to 0\;K\right)$ values.

Due to the PVR, the renormalized measured value to the $1.86\times T_{c}$ (Equations (5) and (8)) (where the latter does not represent the $B_{P}\left(0\right)$ even for aluminum), the PVR does not have any fundamental meaning. To show that the introduction of the renormalization (Equation (8)) is not necessary and experimental $B_{c2,||}\left(T\to 0\;K\right)$ data can be easily described by $\lambda_{e-ph}$ [3], we indicated PVR values in all Figures below, together with $\lambda_{e-ph}$ and other parameters.

For instance, as this was reported in Refs. [5,8,13], magic-angle twisted multilayer graphene (MAT*N*G, *N* = 3, 4, 5) are moderate or strong coupled superconductors. This implies that MAT*N*G (*N* = 3, 4, 5) obeys the following conditions:

(9) $$\left\{\begin{array}{c} 1.0\leq \lambda_{e-ph}\leq 3.0\\ 4.0\leq \frac{2\Delta \left(0\right)}{k_{B}T_{c}}\leq 6.0\\ 1<g\leq 2 \end{array}\right.$$

The limits for parameters in Equation (9) reflect general values observed for major families of superconductors [3,14,15,16]. However, there are some materials which exhibit much higher $\frac{2\Delta \left(0\right)}{k_{B}T_{c}}$ values (see, for instance, Refs. [14,15,17,18]).

If values from Equation (9) substitute in Equation (1), then the inequality of:

(10) $$4\times T_{c}\leq B_{P}{\left(0\right)}_{strong\;coupled}\leq 12\times T_{c}$$

can be easily satisfied. The comparison of Equations (7) and (10) shows that there is no Pauli limit violation in MAT*N*G (*N* = 3,4,5).

## 2. Primary Equation

**Figure 1:**
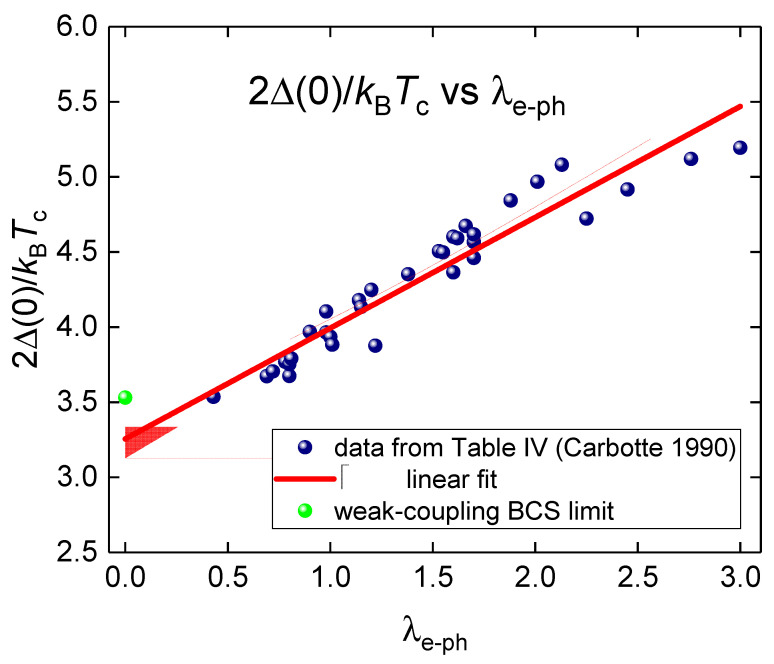
The electron–phonon coupling constant $\lambda_{e-ph}$ vs. the gap-to-transition temperature ratio $\frac{2\cdot \Delta \left(0\right)}{k_{B}\cdot T_{c}}$ (data is taken from Table IV of Reference [3]) and data fit to Equation (10). Deduced parameters are: A=3.26±0.06 and B=0.74±0.04. BCS weak-coupling limit $\frac{2\cdot \Delta \left(0\right)}{k_{B}\cdot T_{c}}=3.53$ is shown by green ball. 95% confidence bands are shown by pink shadow area. Goodness-of-fit is *R* = 0.912.

Equation (1) has two independent parameters, $\Delta \left(0\right)$ and $\lambda_{e-ph}$. To simplify further analysis, it is useful to convert this equation in the form with a single parameter. To make this, we noted that Carbotte [3] collected extended $\frac{2\Delta \left(0\right)}{k_{B}T_{c}}$ vs. $\lambda_{e-ph}$ datasets (Table IV of Reference [3]), which we show in Figure 1. It can be seen (Figure 1) that linear fit provides reasonable accuracy:

(11) $$\left\{\begin{array}{c} \frac{2\Delta \left(0\right)}{k_{B}T_{c}}=A+B\times \lambda_{e-ph}\\ A=3.26\pm 0.06\\ B=0.74\pm 0.04 \end{array}\right.$$

It should be mentioned that there are some approaches that link $\frac{2\Delta \left(0\right)}{k_{B}T_{c}}$ and $\lambda_{e-ph}$ by utilizing more complicated approximating functions [3], or utilized double-valued functions [19] which approximates with better accuracy extended $\frac{2\Delta \left(0\right)}{k_{B}T_{c}}$ vs. $\lambda_{e-ph}$ dataset (included data for hydrogen-rich superconductors [20,21]). However, herein, we used the linear approximate function (Equation (11)) to demonstrate that even this simplest assumption leads to the conclusion that the upper critical field data complies with Pauli limiting field in MAT*N*G. If, at some doping state, the MATNG can exhibit higher $\frac{2\Delta \left(0\right)}{k_{B}T_{c}}$ ratios, then these values will be positioned above the trendline in Figure 1, and, thus, the compliance will satisfy for lower $\lambda_{e-ph}$ values.

Based on all the above, the basic Equation (1) can be approximated by:

(12) $$B_{P}\left(0\right)=\frac{\Delta \left(0\right)}{\sqrt{g}\times \mu_{B}}\times \left(1+\lambda_{e-ph}\right)=\frac{1}{\sqrt{8}}\times \frac{k_{B}}{\mu_{B}}\times \left[\frac{2\Delta \left(0\right)}{k_{B}T_{c}}\right]\times T_{c}\times \left(1+\lambda_{e-ph}\right)\;=\frac{1}{\sqrt{g}}\times \frac{1}{2}\times \frac{k_{B}}{\mu_{B}}\times \left[3.26+0.74\times \lambda_{e-ph}\right]\times T_{c}\times \left(1+\lambda_{e-ph}\right)\;=\frac{1}{\sqrt{g}}\times \left(2.43+2.98\times \lambda_{e-ph}+0.552\times \lambda_{e-ph}^{2}\right)\times T_{c}$$

Equation (12) represents our primary equation which we used for the analysis.

It is useful to demonstrate the difference between widely used Equation (5) and the derived Equation (12) (where, in both equations, g=2 is assumed):

(13) $$\left\{\begin{array}{c} B_{P,BCS}\left(0\right)=1.86\times T_{c}\\ B_{P}\left(0\right)=1.86\times \left(0.92+1.13\times \lambda_{e-ph}+0.21\times \lambda_{e-ph}^{2}\right)\times T_{c} \end{array}\right.$$

It should be mentioned that in the analysis presented herein $B_{c2,||}\left(T\to 0K,\nu ,\frac{D}{\varepsilon_{0}}\right)$ datasets were derived from experimental $B_{c2,||}\left(T,\nu ,\frac{D}{\varepsilon_{0}}\right)$ data by utilizing 10% of the normal state resistance criterion.

## 3. Results

### 3.1. The Pauli Limiting Field Avaluation for Strong-Coupled Superconductors

Due to the issue being equally applied for all superconductors and based on the suggestion of one of the anonymous referees, in Table 1, we showed calculated $B_{p}\left(0\right)$ values for some rounded parameters values for the superconductors exhibited *s*-wave superconducting gap symmetry.

### 3.2. Magic-Angle Twisted Trilayer Graphene (MAT3G)

**Figure 2:**
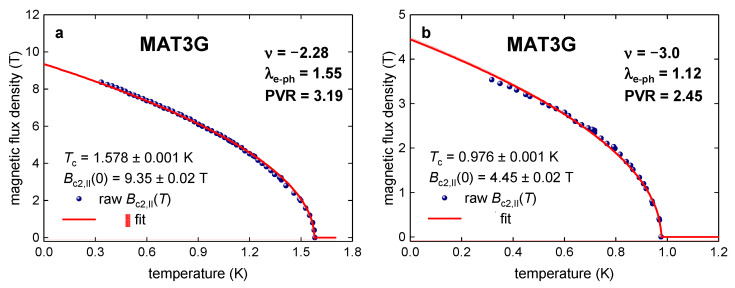
The upper critical field, $B_{c2,||}\left(T,\nu ,\frac{D}{\varepsilon_{0}}\right)$, and the data fit to Ginzburg–Landau expression (Equation (3)) in MAT3G on hole doped side. All measurements are taken at displacement field $\frac{D}{\varepsilon_{0}}=-0.41\;\frac{V}{nm}$. (**a**) $B_{c2,||}\left(T,\nu ,\frac{D}{\varepsilon_{0}}\right)$ at ν = −2.28 and deduced $\lambda_{e-ph}=1.55$ (raw data is from Figure 2b [5]). (**b**) $B_{c2,||}\left(T,\nu ,\frac{D}{\varepsilon_{0}}\right)$ at ν = −3.0 and deduced $\lambda_{e-ph}=1.12$ (raw data is from Figure 2d [5]). PVR values are indicated. The 95% confidence bands are shown by pink shadow area.

**Figure 3:**
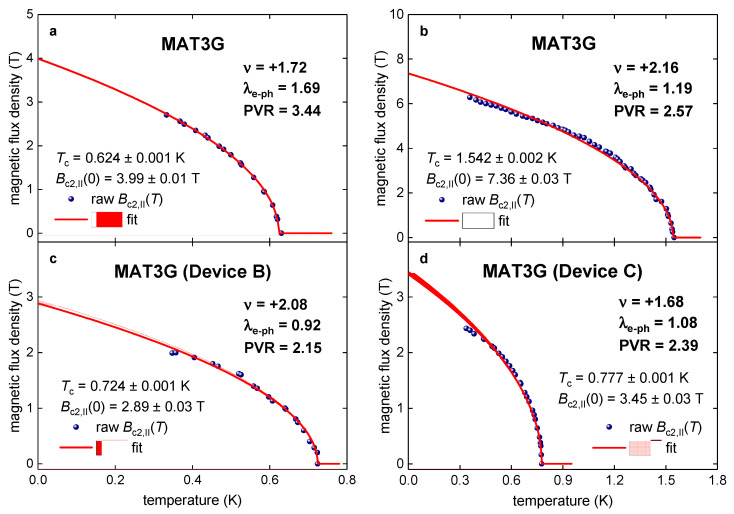
The upper critical field, $B_{c2,||}\left(T,\nu ,\frac{D}{\varepsilon_{0}}\right)$, and the data fit to Ginzburg–Landau expression (Equation (3)) in MAT3G on electron doped side. The data points denote constant-resistance at 10% of the zero-field normal-state resistance. (**a**) $B_{c2,||}\left(T,\nu =1.72,\frac{D}{\varepsilon_{0}}=-0.84\;\frac{V}{nm}\right)$ and deduced $\lambda_{e-ph}=1.72$ (raw data is from Extended Data Figure 1a [5]). (**b**) $B_{c2,||}\left(T,\nu =2.16,\frac{D}{\varepsilon_{0}}=-0.74\;\frac{V}{nm}\right)$ and deduced $\lambda_{e-ph}=1.19$ (raw data is from Extended Data Figure 1b [5]). (**c**) $B_{c2,||}\left(T,\nu =2.08,\frac{D}{\varepsilon_{0}}=0.20\;\frac{V}{nm}\right)$ and deduced $\lambda_{e-ph}=0.92$ (raw data is from Extended Data Figure 2a [5]). (**d**) $B_{c2,||}\left(T,\nu =1.68,\frac{D}{\varepsilon_{0}}=-0.12\;\frac{V}{nm}\right)$ and deduced $\lambda_{e-ph}=1.08$ (raw data is from Extended Data Figure 2b [5]). PVR values are indicated. 95% confidence bands are shown by pink shadow area.

In Figure 2 and Figure 3, we show all $B_{c2,||}\left(T\right)$ datasets for MAT3Gs reported by Cao et al. [5], where we calculate respective $\lambda_{e-ph}$ values based on our primary Equation (11). It can be seen (Figs. 2,3) that all experimental data can be explained by an assumption that MAT3G exhibits very moderate electron–phonon coupling strength, $\lambda_{e-ph}$, close to the one in pure niobium [22]. Thus, there is no necessity to explain extrapolative $B_{c2,||}\left(0\right)$ values in MAT3Gs as a violation of the Pauli limiting field, $B_{p}\left(0\right)$, in these superlattices.

**Figure 4:**
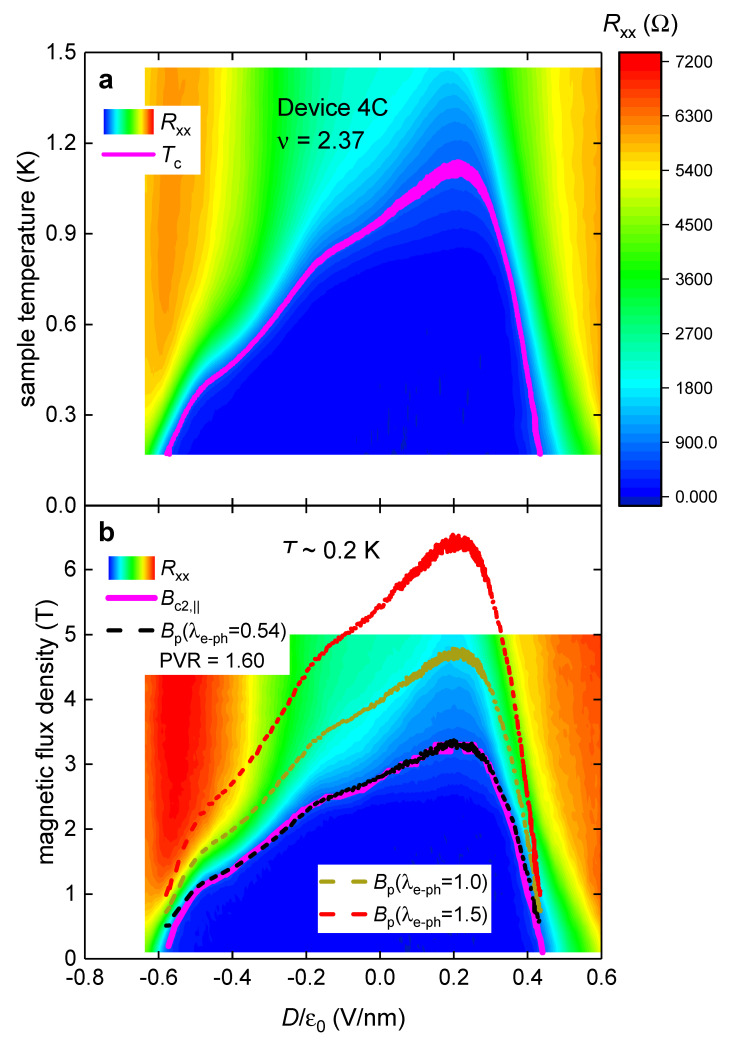
*D*-independent compliance of the Pauli limit field, $B_{p}\left(0\right)$ (Equations (12) and (13)), with the observed upper critical field, $B_{c2,||}\left(T\sim 0.2\;K\right)$, in MAT4G. Raw data is from Extended Data Figure 9c,d in Ref. [8]. $B_{c2,||}$ and $T_{c}$ values were deduced from experimental data by utilizing the resistance criterion of $R_{c}=720\;\pm 40\;\Omega$. (**a**) $R\left(T,\nu ,\frac{D}{\varepsilon_{0}}\right)$ and deduced $T_{c}\left(\nu ,\frac{D}{\varepsilon_{0}}\right)$; (**b**) $R\left(T\sim 0.2\;K,B_{\left|\right|},\nu ,\frac{D}{\varepsilon_{0}}\right)$, deduced $B_{c2,||}\left(T\sim 0.2\;K,\nu ,\frac{D}{\varepsilon_{0}}\right)$, and calculated $B_{p}\left(0,\nu ,\frac{D}{\varepsilon_{0}},\lambda_{e-ph}\right)$ (Equations (12) and (13)). The black curve in (**b**) is plotted for $\lambda_{e-ph}=0.54$ and equivalent PVR value (PVR = 1.60) for this curve is also shown.

**Figure 5:**
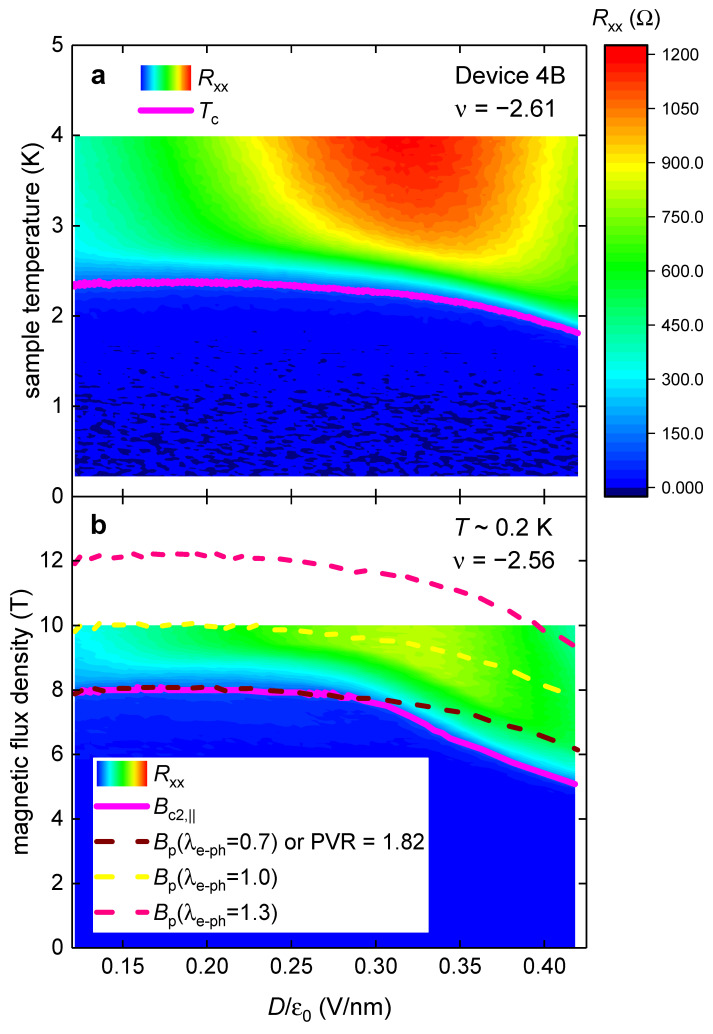
*D*-independent compliance of the Pauli limit field, $B_{p}\left(0\right)$ (Equations (12) and (13)), with the observed upper critical field, $B_{c2,||}\left(T\sim 0.2\;K\right)$, in MAT4G (Device 4B). Raw data is from Extended Data Figure 9a,b in Ref. [8]. $B_{c2,||}$ and $T_{c}$ values were deduced from experimental data by utilizing the resistance criterion of $R_{c}=120\;\pm 10\;\Omega$. (**a**) $R\left(T,\nu ,\frac{D}{\varepsilon_{0}}\right)$ and deduced $T_{c}\left(\nu ,\frac{D}{\varepsilon_{0}}\right)$; (**b**) $R\left(T\sim 0.2\;K,B_{\left|\right|},\nu ,\frac{D}{\varepsilon_{0}}\right)$, deduced $B_{c2,||}\left(T\sim 0.2\;K,\nu ,\frac{D}{\varepsilon_{0}}\right)$, and calculated $B_{p}\left(0,\nu ,\frac{D}{\varepsilon_{0}},\lambda_{e-ph}\right)$. The brown curve in (**b**) is plotted for $\lambda_{e-ph}=0.70$; equivalent PVR value (PVR = 1.82) for this curve is also shown.

**Figure 6:**
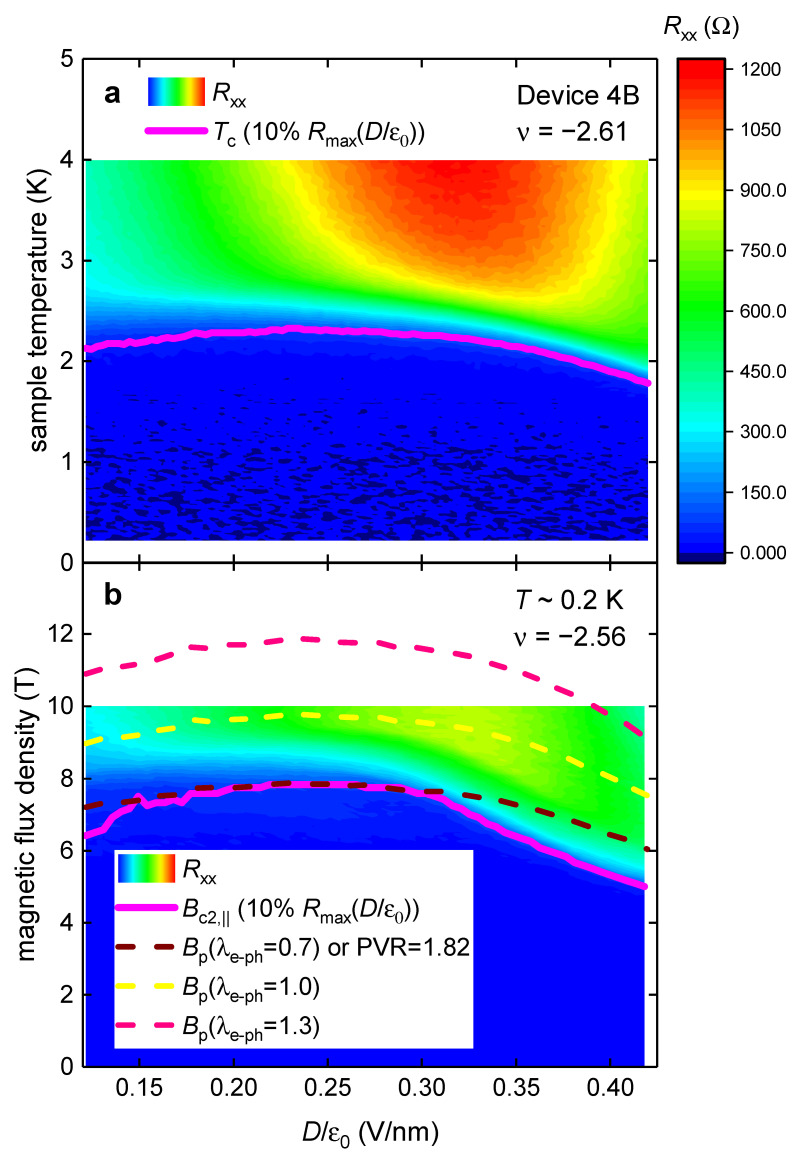
*D*-independent compliance of the Pauli limit field, $B_{p}\left(0\right)$ (Equation (11)), with the observed upper critical field, $B_{c2,||}\left(T\sim 0.2\;K\right)$, in MAT4G (Device 4B). Raw data is from Extended Data Figure 9a,b in Ref. [8]. $B_{c2,||}$ and $T_{c}$ values were deduced from experimental data by utilizing the resistance criterion of 10% of $R\left(T,\nu ,\frac{D}{\varepsilon_{0}}\right)$ at each $\frac{D}{\varepsilon_{0}}$ value. (**a**) $R\left(T,\nu ,\frac{D}{\varepsilon_{0}}\right)$ and deduced $T_{c}\left(\nu ,\frac{D}{\varepsilon_{0}}\right)$; (**b**) $R\left(T\sim 0.2\;K,B_{\left|\right|},\nu ,\frac{D}{\varepsilon_{0}}\right)$, deduced $B_{c2,||}\left(T\sim 0.2\;K,\nu ,\frac{D}{\varepsilon_{0}}\right)$, and calculated $B_{p}\left(0,\nu ,\frac{D}{\varepsilon_{0}},\lambda_{e-ph}\right)$. The brown curve in (**b**) is plotted for $\lambda_{e-ph}=0.70$; equivalent PVR value (PVR = 1.82) for this curve is also shown.

It should be stressed that all indicated $\lambda_{e-ph}$ in Figure 2, Figure 3, Figure 4, Figure 5 and Figure 6 are *minimal* $\lambda_{e-ph}$ values for which the upper critical field complies with the Pauli limiting field. Thus, for any higher $\lambda_{e-ph}$ values, which correspond to the coupling strength realized in the device, the compliance will satisfy with even greater degree.

### 3.3. Magic-Angle Twisted Four-Layer Graphene (MAT4G)

In Figure 4, we show $B_{P}\left(0\right)$ values (calculated by Equation (11)) for magic-angle twisted four-layer graphene Device 4C for which raw data is shown by Park et al. [8] in their Extended Data Figure 9c,d. To deduce $B_{c2,||}$ and $T_{c}$ values from experimental datasets, we utilized the resistance criterion of $R_{c}=720\;\pm 40\;\Omega$ (which is 10% of the maximum resistance measured for this Device 4C).

It can be seen in Figure 4 that the relation of $B_{c2,||}\left(T\sim 0.2\;K,\nu ,\frac{D}{\varepsilon_{0}}\right)≅B_{p}\left(0,\nu ,\frac{D}{\varepsilon_{0}},\lambda_{e-ph}=0.54\right)$ is accurately satisfied across the entire *D*-range. This result implies that there is no Pauli limit violation in this MAT4G film.

It should be stressed, that the $B_{p}\left(0,\nu ,\frac{D}{\varepsilon_{0}},\lambda_{e-ph}\right)$ values (which are overlapped in Figure 4 with $B_{c2,||}\left(T\sim 0.2\;K,\nu ,\frac{D}{\varepsilon_{0}}\right)$) were calculated in the assumption of near weak-coupling electron–phonon interaction, $\lambda_{e-ph}=0.54$. Truly, the latter value is just slightly above the one for pure aluminum $\lambda_{e-ph}=0.43$ [3].

Similar findings (showed in Figure 5) were obtained for the device MAT4G Device 4B (for which the phase diagram is shown by Park et al. [8] in their Extended Data Figure 9a,b). To deduce $B_{c2,||}$ and $T_{c}$ values from experimental data, we utilized the resistance criterion of $R_{c}=120\;\pm 10\;\Omega$ (which is 10% of the maximum resistance measured for this Device 4B). It is important to note that for this device, Park et al. [8] reported raw $R\left(T\sim 0.2\;K,B_{\left|\right|},\nu ,\frac{D}{\varepsilon_{0}}\right)$ data (Extended Data Figure 9a) and raw $R\left(T,\nu ,\frac{D}{\varepsilon_{0}}\right)$ data (Extended Data Figure 9b [8]) which were measured at slightly different filling factor ν=−2.56 (Figure 5a) and ν=−2.61 (Figure 5b). This implies that $T_{c}$ values deduced from the Extended Data Figure 9b (our Figure 5a) are slightly lower than their counterparts expected for the ν=−2.56 filling factor. However, even for this (favorite for the Pauli limiting field violation) choice of raw data, the inequality of $B_{c2,||}\left(T\sim 0.2\;K,\nu ,\frac{D}{\varepsilon_{0}}\right)\leq B_{p}\left(0,\nu ,\frac{D}{\varepsilon_{0}},\lambda_{e-ph}=0.7\right)$ is satisfied across the entire phase diagram (Figure 5).

## 4. Discussion

It should be stressed that above, we calculated $B_{p}$ (Equation (12)) in the assumption that MATNGs exhibit *s*-wave symmetry for the superconducting gap. However, this assumption has not been reaffirmed/disproved in any experiment, and if the MATNGs are *d*- or *p*-wave superconductors, then further increase in calculated $B_{p}\left(0\right)$ (Equation (1)) is expected. This is because the weak-coupling limit for *d*-wave case is $\frac{2\Delta \left(0\right)}{k_{B}T_{c}}=4.28$ [23], and for *p*-wave case $\frac{2\Delta \left(0\right)}{k_{B}T_{c}}=4.06-4.92$ [24] (vs. $\frac{2\Delta \left(0\right)}{k_{B}T_{c}}=3.53$ for *s*-wave [3]). If even MATNGs exhibit *s*-wave gap symmetry, there is a well-established experimental fact that $\frac{2\Delta \left(0\right)}{k_{B}T_{c}}$ in some unconventional *s*-wave superconductors can be as high as $\frac{2\Delta \left(0\right)}{k_{B}T_{c}}\geq 9.0$ [14,15]. In addition, all calculations performed herein were made in the assumption that the Lande factor is g=2. However, the values of g<2 are permitted if material exhibits a reasonable level of spin-orbit scattering. This implies that calculated $B_{p}\left(0\right)$ (Equation (1)) will be further increased.

It is important to mention that recently, Oh et al. [17] and Kim et al. [18] measured the out-of-plane component of the gap, $\Delta_{c}\left(T\right)$, in MATNG (*N* = 2, 3) by scanning tunneling spectroscopy technique. Both research groups [17,18] reported very high gap-to-transition temperature ratios that MATNGs (*N* = 2, 3):

(14) $$15\leq \frac{2\Delta_{c}\left(0\right)}{k_{B}T_{c}}\leq 25$$

The substitution of these $\frac{2\Delta_{c}\left(0\right)}{k_{B}T_{c}}$ limits in Equations (1) and (6) (even in the assumption of the lowest $\lambda_{e-ph}=0.43$, and g=2) leads to very high Pauli limiting field amplitudes, which have never been observed in any experiments on MATNG:

(15) $$9.5\times T_{c}\leq B_{P}{\left(0\right)}_{\lambda_{e-ph}=0;\;g=2}\leq 16\times T_{c}$$

However, it should be mentioned that in considering cases [5,8], the magnetic field is applied to in-plane geometry (i.e., in parallel to the MATNG surface), and for this geometry, the upper critical field depends from the film thickness, $d_{sc}$, and the in-plane coherence length, $\xi_{ab}\left(T\right)$ [25,26,27]:

(16) $$B_{c2,∥}\left(T\right)=\frac{\phi_{0}}{2\pi }\times \frac{1}{\xi_{ab}\left(T\right)}\times \frac{\sqrt{12}}{d_{sc}}$$

Equation (16) can be further converted to the form where $B_{c2,∥}\left(T\right)$ explicitly depends on the in-plane component of the gap, $\Delta_{ab}\left(T\right)$ [7]:

(17) $$\begin{array}{cc} & B_{c2,∥}\left(T\right)=\\ & =\frac{\phi_{0}}{2\pi }\frac{\left(1-0.2429{\left(\frac{T}{T_{c}}\right)}^{2}+0.0396{\left(\frac{T}{T_{c}}\right)}^{4}\right)}{\xi_{ab}\left(0\right)}\frac{\sqrt{12}}{d_{sc}}\sqrt{1-\frac{1}{2k_{B}T}\overset{\infty }{\underset{0}{\int }}\frac{d\varepsilon }{\cosh^{2}\left(\frac{\sqrt{\varepsilon^{2}+\Delta_{ab}^{2}\left(T\right)}}{2k_{B}T}\right)}} \end{array}$$

Considering that the MAT*N*G superlattices are strongly anisotropic, there is an expectation (which was confirmed in recent studies [7]) that the in-plane amplitude of the superconducting gap, $\Delta_{ab}\left(0\right)$, is within the range indicated in Equation (8). However, as we showed above, this level of $\frac{2\Delta_{ab}\left(0\right)}{k_{B}T_{c}}$ ratios in MAT*N*G is enough to satisfy Equations (1) and (2).

In Figure 4 and Figure 5, we defined $B_{c2,||}$ and $T_{c}$ by the criterion of 10% of maximum resistance measured for the device within the full reported $R\left(T,\nu ,\frac{D}{\varepsilon_{0}}\right)$ dataset. To show that a practically identical result can be obtained by implementing other criteria, in Figure 6, we showed the same dataset as in Figure 5, where we implement the criterion of 10% of maximum resistance within each $\frac{D}{\varepsilon_{0}}$ state and reported temperature range. It can be seen in Figure 5 and Figure 6 that both criteria result in identical results.

The approach presented herein can be equally applied if non-electron–phonon-mediated superconductivity can be considered. Truly, for any alternative pairing mechanism, the strength of the non-electron–phonon pairing, $\lambda_{n-e-ph}$, will be presented in respective equations for particular pairing mechanism in regard of Equations (1), (10) and (11). For non-electron–phonon pairing mechanisms the multiplicative pre-factors in these equations can be slightly different from utilized ones. However, because in all considered cases (see Figure 2, Figure 3, Figure 4, Figure 5 and Figure 6), the pairing strength is so moderate (or even close to weak-coupling limit), there is no ground to expect that some exotic pairing mechanism will have so low $\lambda_{n-e-ph}$ that the Pauli limiting field can be violated.

## Figures and Tables

**Table 1:** Calculated Pauli limiting field, $B_{p}\left(0\right)$, for *s*-wave superconductors in accordance with Equations (1) and (10). Values for metals (Al, Sn, and Nb) are included.

$\lambda_{e-ph}$	Landé Factor, *g*	$B_{p}\left(0\right)\;(T)$
$\lambda_{e-ph}=0$; $\frac{2\Delta \left(0\right)}{k_{B}T_{c}}=3.53$ are substituted in Equation (1); Equation (5) is widely used (including [5,8]).	2	$1.86\times T_{c}$
0.43 (Al) [3]	2	$2.7\times T_{c}$
0.72 (Sn) [3]	2	$3.4\times T_{c}$
1.00	2	$4.2\times T_{c}$
1.22 (Nb) [3]	2	$4.9\times T_{c}$
1.50	2	$5.8\times T_{c}$
2.00	2	$7.5\times T_{c}$
2.50	2	$9.4\times T_{c}$
0.43	1.5	$3.1\times T_{c}$
1.00	1.5	$4.9\times T_{c}$
1.50	1.5	$6.7\times T_{c}$
2.00	1.5	$8.7\times T_{c}$
2.50	1.5	$10.9\times T_{c}$
0.43	1.2	$3.5\times T_{c}$
1.00	1.2	$5.4\times T_{c}$
1.50	1.2	$7.4\times T_{c}$
2.00	1.2	$9.7\times T_{c}$
2.50	1.2	$12.2\times T_{c}$

## Data Availability

No new data were created or analyzed in this study. Data sharing is not applicable to this article.
